# Comparative effectiveness of probiotic-based formulations on cecal microbiota modulation in broilers

**DOI:** 10.1371/journal.pone.0225871

**Published:** 2020-05-05

**Authors:** Denise R. Rodrigues, Whitney Briggs, Audrey Duff, Kaylin Chasser, Raj Murugesan, Chasity Pender, Shelby Ramirez, Luis Valenzuela, Lisa R. Bielke

**Affiliations:** 1 Department of Animal Sciences, The Ohio State University, Columbus, Ohio, United States of America; 2 BIOMIN America Inc., Overland Park, Kansas, United States of America; 3 BIOMIN Holding GmbH, Getzersdorf, Austria; USDA-Agricultural Research Service, UNITED STATES

## Abstract

The potential of probiotics to manipulate the intestinal microbial ecosystem toward commensal bacteria growth offers great opportunity for enhancing health and performance in poultry. This study aimed to evaluate the efficacy of five probiotic-based formulations in modulating cecal microbiota in broilers at 21 and 42 days of age. Probiotics investigated included a synbiotic (SYNBIO), a yeast (YEAST), and three single-strain formulations of *Bacillus amyloliquefaciens* (SINGLE1), *B*. *subtilis* (SINGLE2) and *B*. *licheniformis* (SINGLE3). Alpha-diversity analyses showed that cecal microbiota of SINGLE1, SINGLE2, and YEAST had low diversity compared to the control diet with no feed additive (CON) at 21d. At the same age, weighted Unifrac distance measure showed significant differences between samples from SYNBIO and CON (*P* = 0.02). However, by analyzing principal coordinates analysis (PCoA) with unweighted Unifrac, there was no evidence of clustering between CON and probiotic treatments. By 42d, there were no differences in alpha or beta-diversity in the microbiota of probiotic treatments compared to CON. Similarly, taxonomic microbial profiling did not show major changes in cecal microbial taxa. In conclusion, not all probiotic-based formulations tested had a core benefit on the modulation of microbiota. However, based on the quantitative beta diversity results, SYNBIO greatly influenced the cecal microbial community structure attributable to transient variations in relative taxon abundance.

## Introduction

The balance between the host immune system and intestinal microbial community plays an essential role in health and disease. While pathogen-induced microbiota disruption has been associated with many intestinal and systemic conditions, beneficial bacteria colonization is often linked to high productivity in broilers [1–3]. Therefore, it is becoming increasingly clear that manipulating the microbiota of the gastrointestinal tract (GIT) can be an effective strategy to stimulate a healthy balanced microbial community as a means of improving overall health and performance in poultry [4,5].

In this context, probiotics have been identified as a promising nutritional intervention to promote modulation of GIT microbiota toward commensal bacteria growth [5–8]. However, modification of the microbial population is not considered a general benefit for probiotic supplementation [9]. Mechanisms such as competitive exclusion are widespread among probiotic formulations[10]. Nevertheless, effects at the intestinal level are more likely to be strain-specific.

Although several bacterial species and yeasts from *Bacillus*, *Lactobacillus*, *Enterococcus*, *Bifidobacterium*, *Pediococcus*, and *Saccharomyces* genera have been described as probiotic for broiler chickens [5,11], the probiotic features are more specific to the selected strain than the genus of origin [10]. Additionally, studies with mouse models have shown that taxonomically similar probiotic species produced by different manufacturing methods can exert divergent effects on disease attenuation [12].

Synbiotic supplementation has drawn recent attention owing to the potential for modification of the gut microbiota and its metabolites [11,13,14]. Synbiotics have both probiotic and prebiotic properties. Prebiotics are food components, usual carbohydrates of various molecular structures that are not digestible by the host, and can be selectively fermented by potentially beneficial bacteria [13]. Since prebiotics are used mostly as a selective medium for the growth of probiotic, the alterations in the intestinal microbial community may occur at the level of individual strains and species [13].

Scientific advances in the field of microbiology have provided crucial insights into the mode of probiotic action. For instance, 16S rRNA and metagenomic analyses have maximized the knowledge about microbial communities and contributed to developing microbiota-based probiotics. However, there have been inconsistencies concerning the response of probiotic supplementation on the modulation of GIT microbial communities, which underlines the need for a more thorough comprehension of the mechanisms by which probiotics influence the microbiota.

In order to achieve a better understanding of how different probiotic mixtures can affect the GIT microbiota composition in broiler chickens, this study was conducted to evaluate the efficacy of five probiotic-based formulations in modulating diversity and relative abundance of cecal microbial communities in 21 and 42-day-old broilers.

## Material and methods

### Experimental design and dietary treatments

A total of 720 one-day-old Ross 708 male chicks were allocated to 6 treatments in a completely randomized design. Eight replicates were assigned to each of the treatments with 15 birds per replicate. Treatments were based on supplemental diets including (1) basal diet no treatment (CON); (2) Synbiotic (0.45 g/Kg; SYNBIO); (3) Yeast-based probiotic (1.12 g/Kg; YEAST); (4) Single-strain probiotic 1 (0.45 g/Kg; SINGLE1); (5) Single-strain probiotic 2 (0.27 g/Kg; SINGLE2) or (6) Single-strain probiotic 3 (0.45 g/Kg; SINGLE3). The broilers were continuously fed treated diets throughout the trial.

The SYNBIO-based mixture was composed of 2 × 10^11^ CFU/g multi-species probiotic, including *Lactobacillus reuteri*, *Enterococcus faecium*, *Bifidobacterium animalis*, *Pediococcus acidilactici*, and a prebiotic (fructooligosaccharide). The formulation YEAST was a probiotic-containing *Saccharomyces cerevisiae* (Moisture 11%, Crude fiber 25%). The single-strain probiotics were composed of spore-forming *Bacillus* spp. The formulation SINGLE1 contained 1.25 × 10^6^ CFU/g of *B*. *amyloliquefaciens*, while SINGLE2 comprised 10 billion spores/g of *B*. *subtilis*. Besides, each gram of the SINGLE3 contained 3.20 ×10^9^ CFU of *B*. *licheniformis*.

Birds were reared from 1 to 42d and housed in floor pens on fresh wood shavings litter with *ad libitum* access to a standard corn-soy diet and water (S1 Table) [15]. From 1 to 7 days of age, chicks were exposed to 23 hours of light and a 1-hour dark lighting program. After 7 days, a 1 hour period of darkness was increased every week. The feeding program consisted of 3 phases: starter (1-7d), grower (8-21d), and finisher (22-42d). Stater diets were in mash form, whereas the grower and finisher diets were pelleted. All experimental procedures were approved by the Ohio State University’s Institutional Animal Care and Use Committee (IACUC).

### Sample collection and processing

We selected four birds per pen to investigate the intestinal microbiota composition of probiotic-treated broilers on days 21 and 42. Post-euthanasia, the samples from cecal contents were collected, immediately immersed in liquid nitrogen and kept frozen at -18°C until further DNA extraction. Cecal contents were weighed and mixed to create pooled samples from two birds (n = 16 per treatment for each time collection) for DNA extraction. Next, 0.3 g of the mixed digesta was added into a 2.0 mL screwcap microcentrifuge tube with 0.2 g of zirconia beads (0.1 mm). DNA was extracted from each sample, along with pure culture bacterial samples, using the protocol from Arthur et al. [16] with several modifications. In brief, phenol: chloroform: iso-amyl alcohol (25:24:1, 1 phase) was used for all DNA washings, during which the extraction sample supernatant was mixed with 500 μL of the phenol: chloroform: iso-amyl alcohol. After adding Buffer AL (Qiagen, Germantown, Maryland) and ethanol, samples were placed on to EconoSpin Silica Membrane Mini spin columns (Epoch Life Science Inc., Missouri City, TX, USA) and centrifuged (14,000 rpm at 21°C) for the same time durations rather than placed onto a vacuum manifold. After extractions were completed, DNA quality and quantity were measured using a Synergy HTX, Multi-Mode Reader (BioTek, Winooski, VT), and all samples were diluted to a concentration of 20 ng/μL for 16S rRNA sequencing analysis.

### 16S rRNA library preparation and sequencing methods

High quality RNase-treated genomic DNA was submitted to the Molecular and Cellular Imaging Center (MCIC, http://mcic.osu.edu/home) in Wooster, Ohio, for library preparation. The DNA samples were quantified and normalized before library preparation. The V4 hypervariable region of the bacterial 16S rRNA gene was targeted in this study. To amplify and sequence the region of interest, we used primers that contain a heterogeneity spacer in line with the targeted sequence. Four sets of spacers of different lengths were used to compensate for the low nucleotide diversity of the amplicons; since accurate base-calling on Illumina platforms and generation of high-quality data requires sequence diversity at each nucleotide position before the clustering occurs. For the targeted region, we used 515F and 806R primers (515F: GTGYCAGCMGCCGCGGTAA, 806R: GGACTACHVGGGTWTCTAAT), which include degenerate bases for maximal inclusiveness [17].

Libraries were prepared in two rounds of PCR amplification. The first round amplified the locus of interest and added a portion of the Illumina adapter sequence. The second round completed the Illumina adapter sequence, which contained a unique dual combination of the Nextera indices for individual tagging of each sample. Twenty-five nanograms of each genomic DNA were used as input for the first PCR reaction, and 3 uL of the clean PCR 1 product was used as input for the second PCR reaction. PCR amplifications were carried as follows: initial denaturation at 96°C for 3 min, followed by 25 (PCR 1) or 8 (PCR 2) cycles each of 96°C for 30 s, 55°C for 30 s and 72°C for 30 s, and a final extension at 72°C for 5 min. The PCR products were purified after each PCR amplification using the Agencourt AMPure XP beads (Beckman Coulter Life Sciences). All the steps for library preparation and cleaning were carried out on the epMotion5075 automated liquid handler (Eppendorf). The purified amplicon libraries were quantified and pooled at equimolar ratios before sequencing. The final pool was validated for size and absence of primer dimers on the TapeStation 4200 system (Agilent) and quantified using Qubit 2.0 fluorometer system (ThermoFisher Scientific).

The amplicon libraries were using the MiSeq sequencing platform (Illumina) at a final concentration of 14.3 pM. PhiX was mixed in with the pool of amplicon libraries for the sequencing run (expected at 20%). The run was clustered to a density of 771 k/mm2, and the libraries were sequenced using a 300PE MiSeq sequencing kit with the standard Illumina sequencing primers. Image analysis, base calling, and initial data quality assessment were performed on the MiSeq instrument.

### Bioinformatics processing

Sequencing quality screen was performed to ensure high-quality sequences. Briefly stated, sequence quality was determined using the FASTQC, and MultiQC toolkits [18] Sequence reads exhibiting a quality score of lower than 20 were removed. Further, low complexity reads, those shorter than 200 bp in length, and mismatched primers were also eliminated. Additionally, reads exhibiting low sequence qualities on either end were trimmed. The pre-processed FASTQ files were then imported to the QIIME2 2019.7 [19] platform for analysis. The main analytical steps were as follows: firstly, reads were de-multiplexed and classified into their respective samples. Next, additional sequence quality control measures and feature table construction were performed by the DADA2 algorithm [20].

Shannon’s diversity index and pairwise UniFrac distances [21] were estimated using q2-diversity in QIIME2. Taxonomy was assigned to amplicon sequence variants (ASVs) using the q2-feature-classifier plugin against the Greengenes 13_8 99% OTUs reference sequences database [22]. The sequencing datasets for this study are available at Sequence Read Archive under BioProject accession number PRJNA578362.

### Statistical analysis

Kruskal-Wallis pairwise test was assessed to compare differences in the microbial Shannon’s diversity index (H) across treatments. Weighted Unifrac distance metric was used for comparing the cecal beta-diversity between probiotic and non-treated samples (PERMANOVA, *P* ≤0.05). Principal coordinates analysis (PCoA) of unweighted Unifrac distance were addressed to measure the similarity between cecal samples. The mean relative abundances of microbial communities were compared by ANOVA, followed by the Tukey post hoc test (*P*≤0.05) using the JMP Pro13 Software (JMP Software, SAS Inc., 2018). For the microbiome plots and heat maps, we used Rstudio software (Version 1.1.463, 2009–2018 RStudio, Inc.).

## Results

Diversity and composition of cecal microbiome in broilers supplemented with different probiotic formulations were assessed based on 16S rRNA sequencing. A total of 5,348,269 16S rRNA raw sequence reads were obtained. The number of sequencing reads of overall samples ranged from 13,545 to 60,125 with a mean of 27,855.82.

### Microbiome diversity measures

**Fig 1:**
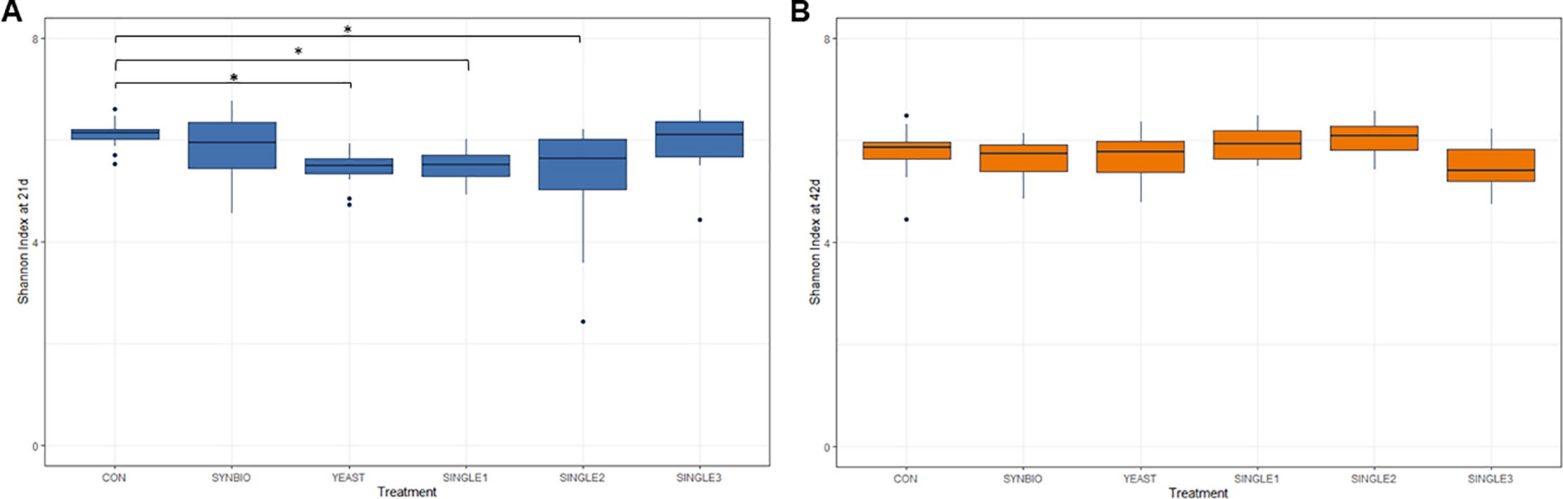
Alpha-diversity in cecal microbiota of broiler chickens treated with different probiotic formulations. (A) Box plots showing the Shannon index of microbial communities sampled in 21 day-old broilers fed a basal diet with no treatment (CON), synbiotic (SYNBIO), yeast-based probiotic (YEAST) or single-strain formulations composed of *Bacillus amyloliquefaciens* (SINGLE1), *B*. *subtilis* (SINGLE2), and *B*. *licheniformis* (SINGLE3). Panel (B) illustrates the diversity index of microbiota in ceca of 42-days-old broilers.

Cecal microbiota alpha-diversity was compared between CON and probiotic-treated groups at 21d (Fig 1A) and 42d (Fig 1B). Shannon’s diversity index indicated that the ceca of SINGLE1, SINGLE2, and YEAST supported a less diverse microbial community comparable with CON at 21d (*P*<0.05). On day 42, there were no significant differences in the Shannon index compared to CON.

We used two approaches to measure the beta-diversity of cecal microbial community: weighted UniFrac, a quantitative measure which detects changes in the relative abundance of present taxa, and unweighted UniFrac, a qualitative test which use the presence/absence of sequencing data to compare the community composition.

**Fig 2:**
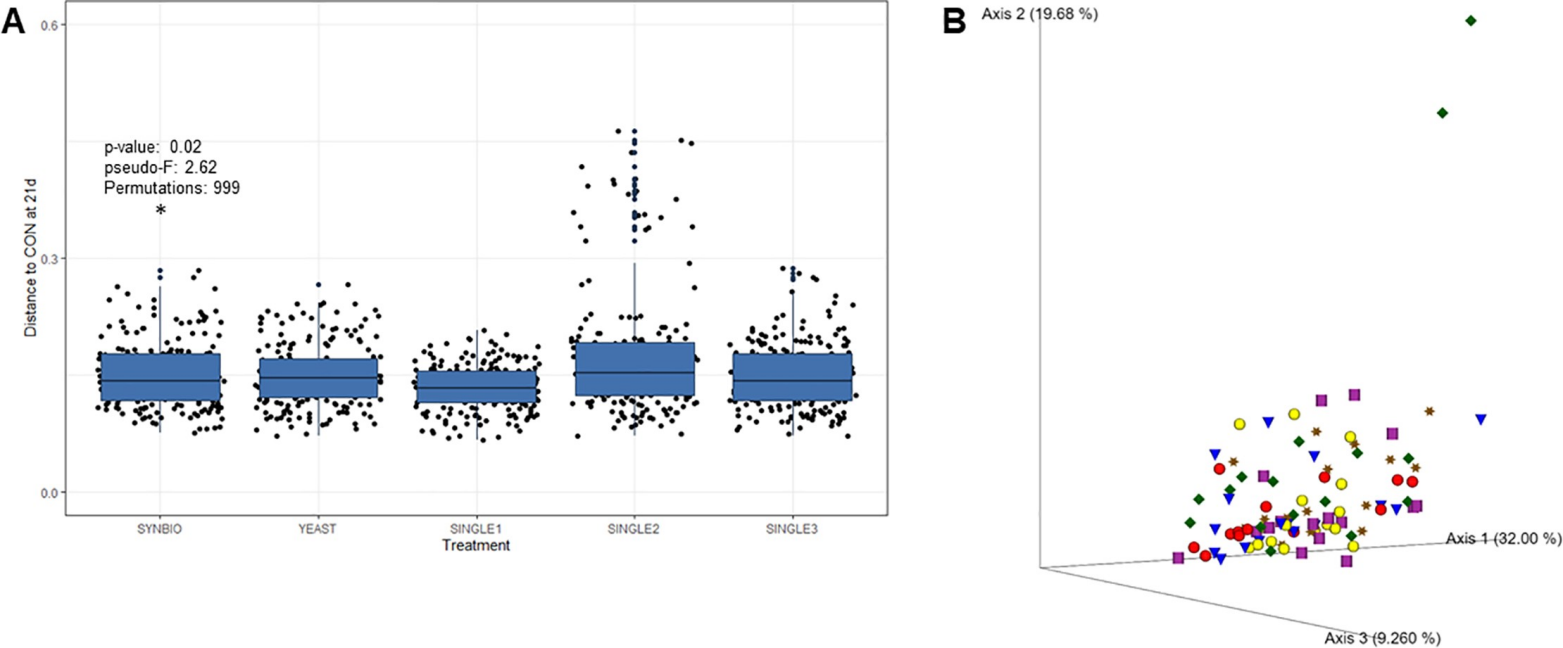
Quantitative and qualitative diversity measures of cecal microbiota in broilers at 21 days of age. (A) Pairwise comparison based on weighted Unifrac distances between cecal microbial communities from broilers fed a basal diet with no treatment (CON), synbiotic (SYNBIO), yeast-based probiotic (YEAST), or single-strain formulations composed of *Bacillus amyloliquefaciens* (SINGLE1), *B*. *subtilis* (SINGLE2), and *B*. *licheniformis* (SINGLE3) by 21d. (B) Principal coordinate analyses plot of cecal samples derived from unweighted UniFrac (CON- red sphere, SYNBIO- dark orange star, YEAST- purple cylinder, SINGLE1- yellow sphere, SINGLE2- green diamond, and SINGLE3- blue cone).

Weighted pairwise Unifrac distance metric, using the Pseudo-F test, showed significant differences between samples from SYNBIO and CON (*P =* 0.02, pseudo-F: 2.62, Permutations: 999) at 21d (Fig 2A; S2 Table). However, by analyzing principal coordinates analysis (PCoA) of cecal samples with unweighted Unifrac, there was not evident clustering between CON and probiotic treatments at the same age (Fig 2B). These results suggest that although the addition of SYNBIO in the feed did not contribute to affect the occurrence of cecal bacterial lineages, it may have influenced the relative abundance of present microbial communities. It is also worthy of highlighting that the weighted UniFrac difference was observed only by 21 days of age, indicating that supplementation of SYNBIO is related to transient changes in microbial communities.

**Fig 3:**
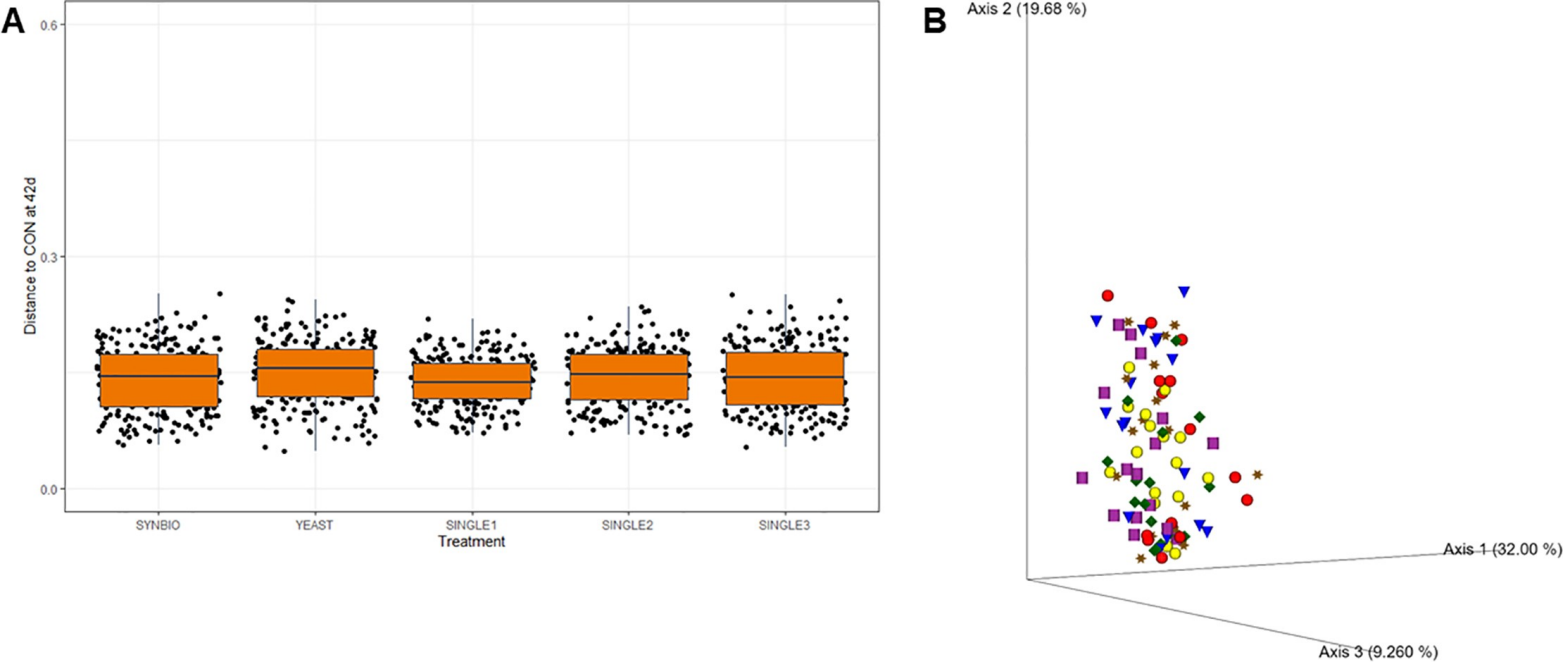
Weighted and unweighted UniFrac measurements of cecal microbiota in broilers at 42 days of age. (A) Pairwise comparison based on weighted Unifrac distances between cecal microbial communities. (B) Principal coordinate analyses plot based on unweighted UniFrac showing similarity of cecal samples from broilers fed a basal diet with no treatment (CON- red sphere), synbiotic (SYNBIO- dark orange star), yeast-based probiotic (YEAST- purple cylinder), or single-strain formulations composed of *Bacillus amyloliquefaciens* (SINGLE1- yellow sphere), *B*. *subtilis* (SINGLE2- green diamond), and *B*. *licheniformis* (SINGLE3- blue cone) by 42 days of age.

By 42 days of age, there were no significant changes in the weighted pairwise Unifrac distance metric (Fig 3A). Correspondingly, PCoA plots indicate a similarity of the consortia derived from the non-treated and probiotic-treated cecal samples (Fig 3B).

**Fig 4:**
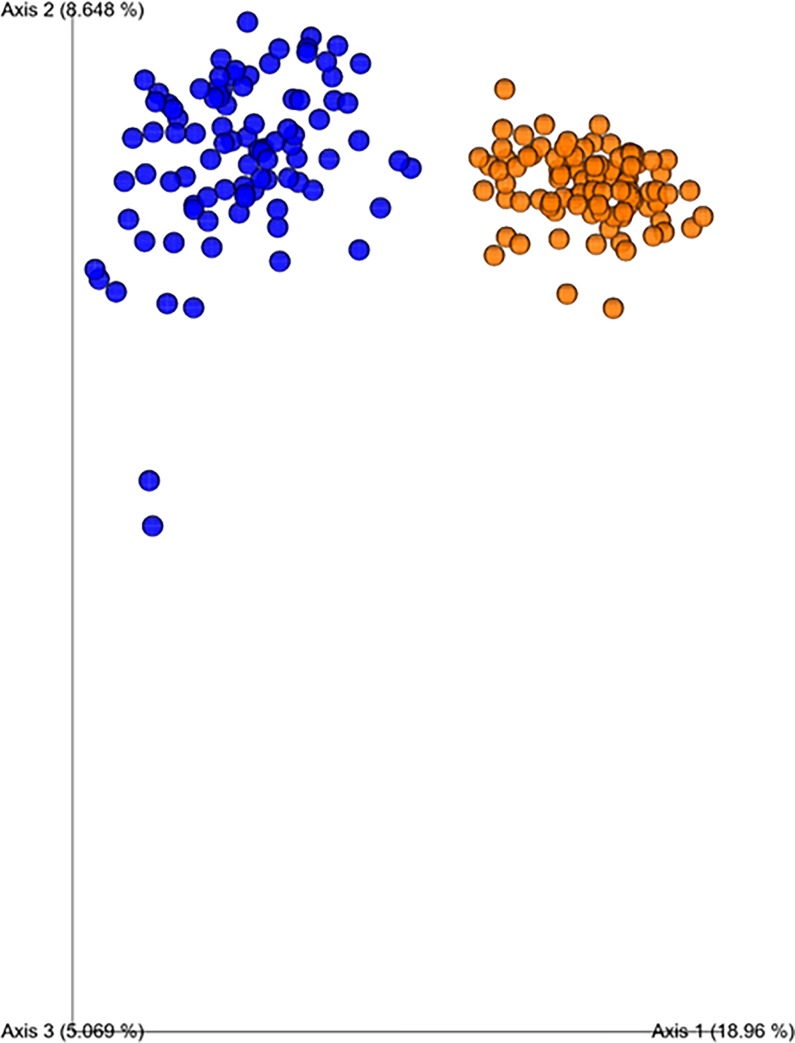
Principal coordinate analyses plot using unweighted UniFrac confirmed bacterial community differences centered on the bird’s age. (*P*<0.05, PERMANOVA, blue sphere = 21d, orange sphere = 42d).

Furthermore, as shown in Fig 4, there was a clustering of samples based on the birds' age. The PCoA plots illustrate the predominant role of age in driving microbiome composition regardless of the probiotic supplementation effect. This dissimilarity between samples from 21 and 42 day-old broilers, revealed by unweighted metric analyses, is further supported by our taxonomic-based analyses.

### Microbial community composition

**Fig 5:**
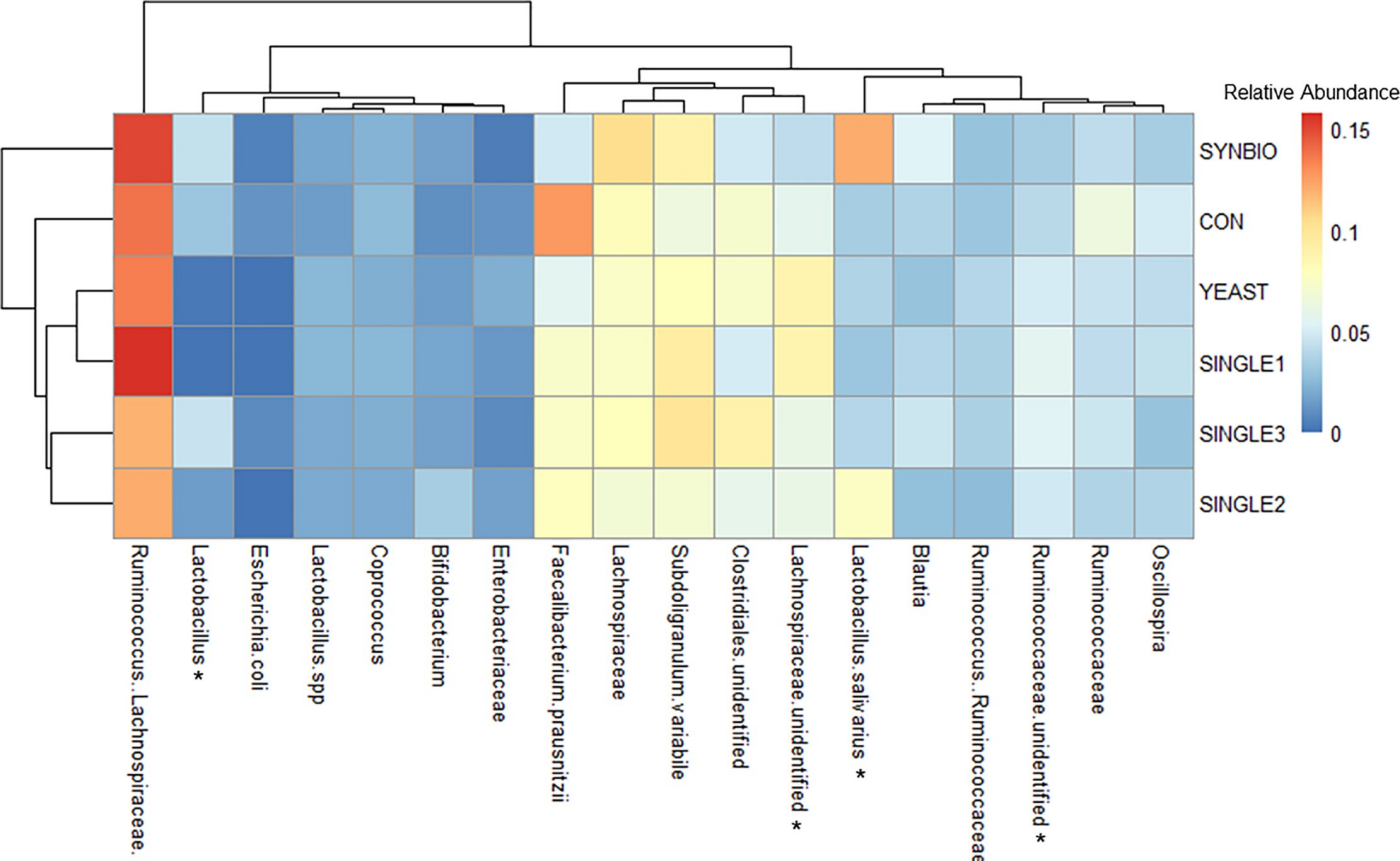
Double hierarchical dendrogram and heat map of cecal bacterial communities from 21-day-old broilers. Heatmap plot represents the relative abundance changes of the most predominant microbial families, genera, and species in the ceca. Hierarchical clustering in the rows is based on the composition similarity between treatments, while that in the columns is based on the microbial relative abundances closeness. Statistical differences (*P*<0.05) between groups were reported for each bacterial population (*).

**Fig 6:**
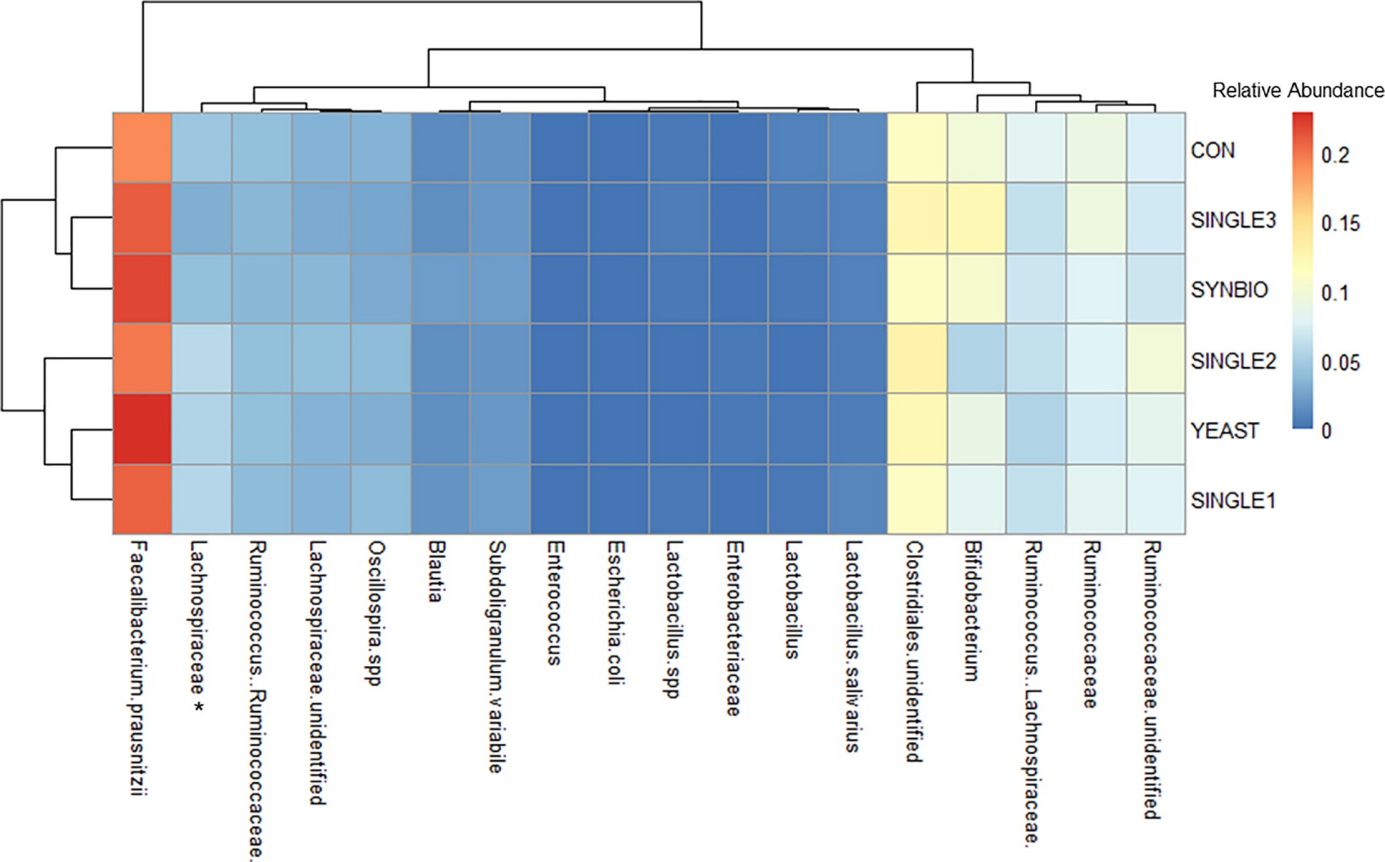
Clustered heat map based upon the predominant bacterial families, genera, and species identified in the ceca microbiome of 42-day-old broilers treated with different probiotic formulations. Hierarchical clustering in the rows is based on the composition similarity between treatments, while that in the columns is based on the microbial relative abundances closeness. Statistical differences (*P*<0.05) between groups were reported for each bacterial population (*). Diet with no treatment (CON), supplementation with synbiotic (SYNBIO), yeast-based probiotic (YEAST), and single-strain formulations composed of *Bacillus amyloliquefaciens* (SINGLE1), *B*. *subtilis* (SINGLE2), and *B*. *licheniformis* (SINGLE3).

To identify the impact of different probiotic formulations on cecal microbiome makeup, we analyzed the16S rRNA taxonomic microbial profiling. Interestingly, none of the bacterial strains supplemented in the feed were detected on the taxonomic profiling from the cecal samples by 21 or 42 days of age. As displayed in Figs 5 and 6, the addition of probiotic-based feed did not result in major changes in cecal microbial taxa related to CON group.

In this study, more than 99.90% of bacterial rDNA sequences were assigned to the domain Bacteria. Dominant bacterial families found in ceca belonged to Clostridiales. Regarding these families, SYNBIO had the greatest (*P*>0.05) population of *Lachnospiraceae*, even though the relative abundance of unidentified *Lachnospiraceae* was significantly reduced compared to SINGLE1. Additionally, the supplementation of SINGLE3 significantly increased unidentified *Ruminococcaceae* in ceca compared to CON and SYNBIO at 21d (S3 Table).

Changes in abundance of genus and species from Lactobacillales were also seen between treatments by 21d. The addition of SYNBIO in feed changed the cecal microbiota composition of broilers by increasing (*P*<0.05) the population of *Enterococcus* compared to SINGLE1, SINGLE2, and YEAST (S3 Table). It should be pointed out that one of the probiotic strains contained in SYNBIO mixture belongs to *Enterococcus* genus. *Lactobacillus salivarius* was also increased in SYNBIO relative to SINGLE1, while YEAST and SINGLE1 reduced (*P*<0.05) the percentage of *Lactobacillus* compared to SINGLE3 and SYNBIO.

By 42 days of age, there were no broad influences of probiotic supplementation on the cecal microbial profile, although a microbial succession pattern was evident (Figs 5 and 6). A considerable increase in *Bifidobacterium* and *Faecalibacterium prausnitzii* populations were detected. Nonetheless, there were no significant shifts across treatments when compared to CON. Notably, the *Lachnospiraceae* had a higher (*P*<0.05) population in SINGLE1, SINGLE2, and YEAST than SNGLE3 and SYNBIO.

## Discussion

In this study, we investigated the impact of different probiotics in modulating the composition and structure microbial diversity of cecal bacterial communities in 21 and 42-day-old broilers. We found that the tested probiotic mixtures differently affected the richness and evenness of cecal microbial communities in young broilers. Based on alpha diversity measurements, we identified that supplementation of SINGLE1, SINGLE2, and YEAST reduced the diversity, whereas SYNBIO and SINGLE3 had similar Shannon index compared to CON. These results disagreed with Wang [23], who reported that supplementation of probiotics in the feed promoted higher biodiversity of the intestinal microbiome in poultry. However, as excellently reviewed by Reese and Dunn [24], livestock can have high performance with low-diverse GIT microbiota. This prediction is supported by the notion that the host immune system may limit microbial diversity, given that not all microbes are beneficial [25]. Besides, the overabundance of commensal bacteria may lead to a low level of diversity in the intestinal ecosystem [8]. Nevertheless, stress conditions or GIT pathogen colonization can induce a reduction of microbial diversity in poultry [26,27]. The loss in diversity driven by a dysbiotic microbiota is found with a carriage of commensals such as lactobacilli reduced, while the level of *Enterobacteriaceae* increased [28,29]. Here, in this study, there was no pathogen challenge or evident environmental stress imposed on the broilers during the experimental period.

Investigations concerning GIT microbiome diversity and composition have emerged due to evidence that microbiota manipulation may benefit host metabolism, performance, and immune protection to diseases [30–32]. Given that modulation of microbiota can be driven by genetics, diet, environmental conditions, and intestinal pioneer colonization [8,33,34], the age and physiology of organs are identified to play a primary role in influencing composition and diversity of GIT bacterial populations [24,26]. Indeed, ceca have an important function in fermentation and are well known to be the most diverse GIT organ in birds, with a predominance of Clostridiales members [35]. It has been reported by Lu et al. [35] that the microbial community structure is fairly stable during periods of rapid skeletal growth. For instance, our data showed that there was an age-related difference between 21d to 42d microbiomes, in which microbial shifts were centered on species belonging to *Lactobacillaceae* and *Bifidobacteriaceae*, followed by *Faecalibacterium prausnitzii*.

The results suggest that the different probiotic formulations did not elicit significant changes in the cecal microbiota populations relative to CON. As observed in the taxonomic profiling at 21 days of age, the supplementation of SINGLE3 significantly increased the unidentified *Ruminococcaceae* population in ceca, while YEAST and SINGLE1 fed broilers had a lower abundance of *Lactobacillus* compared to SINGLE3 and SYNBIO. In contrast with our results, Bortoluzzi [36] reported that supplementation of *Saccharomyces cerevisiae*-based probiotic did not alter the cecal counts of *Lactobacillus* and *Enterococcus* compared to a basal diet. Following this study, Ma et al. [7] reported that the addition of *Bacillus*-based probiotics affected the cecal microbial composition in broilers by reducing members of *Lactobacillus* along with *Ruminococcaceae*.

The phylogenetic distances using UniFrac revealed that the supplementation of SYNBIO affected a quantitative measure of beta microbial diversity by 21 days of age. This finding indicates a distinction in the SYNBIO microbial community structure relative to CON due to changes in relative taxon abundance. As reported by Lozupone et al. [21], differences in abundance for a particular set of taxa may happen as a result of the nutrient availability in the ecosystem. Of relevance, the supplementation of SYNBIO resulted in a modulation of intestinal microbiota with a greater relative abundance of *Lachnospiraceae* and *Lactobacillus salivarus*, which may explain the differences found in the bacterial community presented by weighted Unifrac measure. Consistently, the hierarchical cluster analysis showed that SYNBIO samples displayed a unique microbial composition with a separation from the other probiotic treatments by 21d (Fig 5). However, these notable differences of the SYNBIO microbial community did not persist through 42d. Probiotics seem to have the greatest effect during the initial development of the microbiota [37]. The heightened potential of symbiotic formulations is accredited to mechanisms shared by both probiotics and prebiotics. Teng and Kim [38] have reported that prebiotics from the inulin group might stimulate the growth and activity of beneficial bacteria by increasing the concentration of short-chain fatty acids and lactic acid in the ceca of broilers. It is worth highlighting that the intestinal colonization of LAB in poultry has been associated with a reduction of pathogens, higher performance, and development of the immune system [8,14,39,40]. A higher settlement of LAB in the GIT may also lead to enhanced energy and mineral recovery from nutrients and result in better digestive efficiency [41,42].

Based on our results, we conclude that not all probiotic-based formulations tested here had a core benefit on the modulation of microbiota. Relying on the quantitative beta diversity results, SYNBIO greatly influenced on the cecal microbial community of 21-day-old broilers attributable to variations in relative taxon abundance. This finding suggests the dietary supplementation of SYNBIO might be associated with transient factors within the cecal ecosystem. Therefore, prospective studies are warranted to identify associations between probiotic formulations and microbial resource availability as a means of increasing our understanding of how microbial-based interventions could offer effective opportunities to shape the GIT microbial communities in broiler chickens.

## Supporting information

S1 Table Pairwise comparison based on weighted Unifrac distances between cecal microbial communities from broilers. (XLSX)

S2 Table Taxonomy of cecal microbial communities in broilers at 21 and 42 days of age. (XLSX)

S3 Table Taxonomy of the predominant cecal microbial communities. (XLSX)
